# Supplementary material for: Mapping of mitogen and metabolic sensitivity in organoids defines requirements for human hepatocyte growth
Source: Nat Commun. 2024 May 13;15:4034. doi: 10.1038/s41467-024-48550-4 (PMC11091073; doi:10.1038/s41467-024-48550-4)
Supplement: Supplementary file 1 — Supplementary Information [file 41467_2024_48550_MOESM1_ESM.pdf]

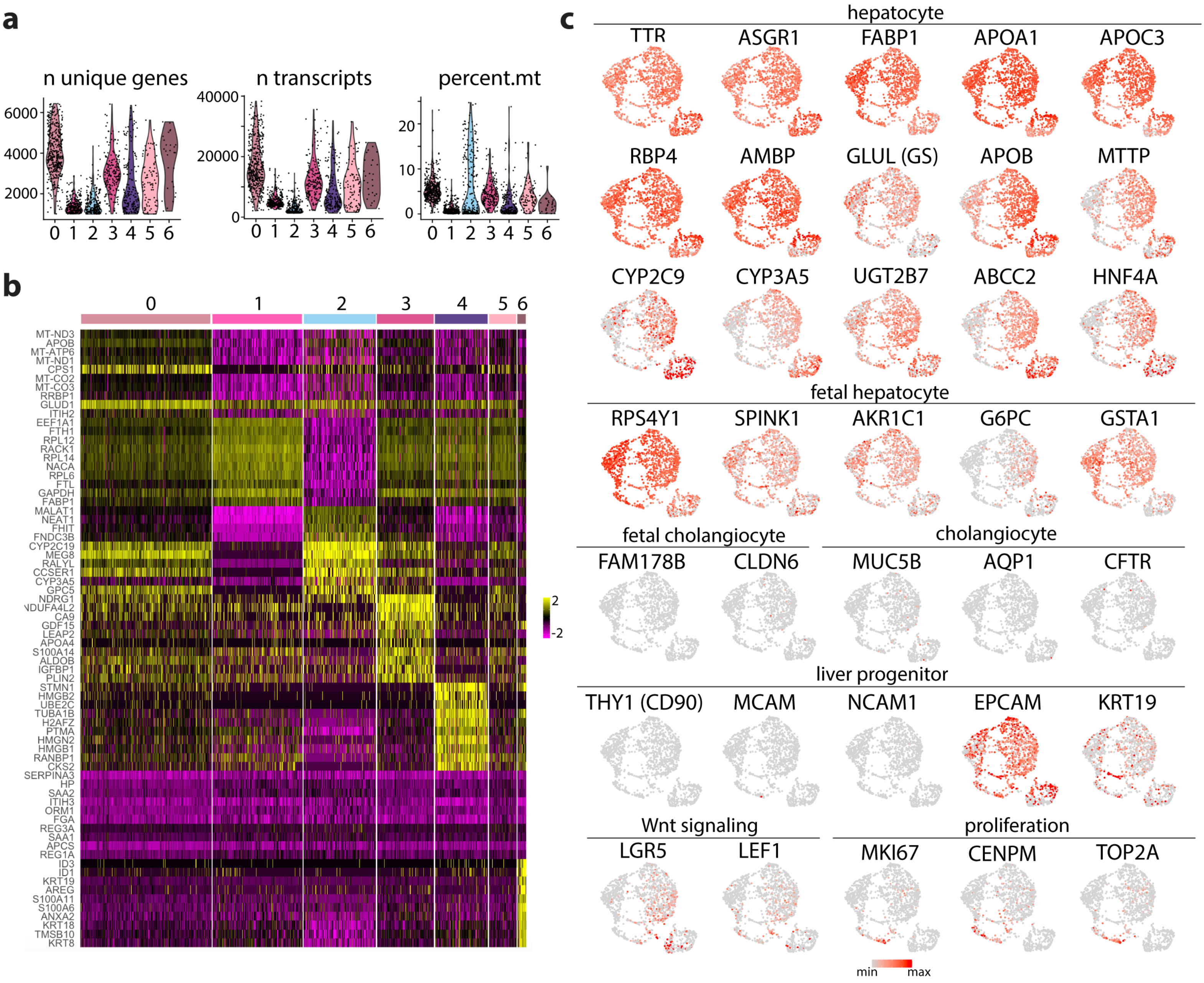

Supplementary Figure 1. Single-cell RNA sequencing analysis of human fetal hepatocyte organoids.
a, Data quality measures across the different FH organoid clusters (see **Fig. 2a**).
b, Heatmap visualizing gene expression trends across clusters of the top 10 genes per cluster.
c, UMAP plots of the indicated markers.

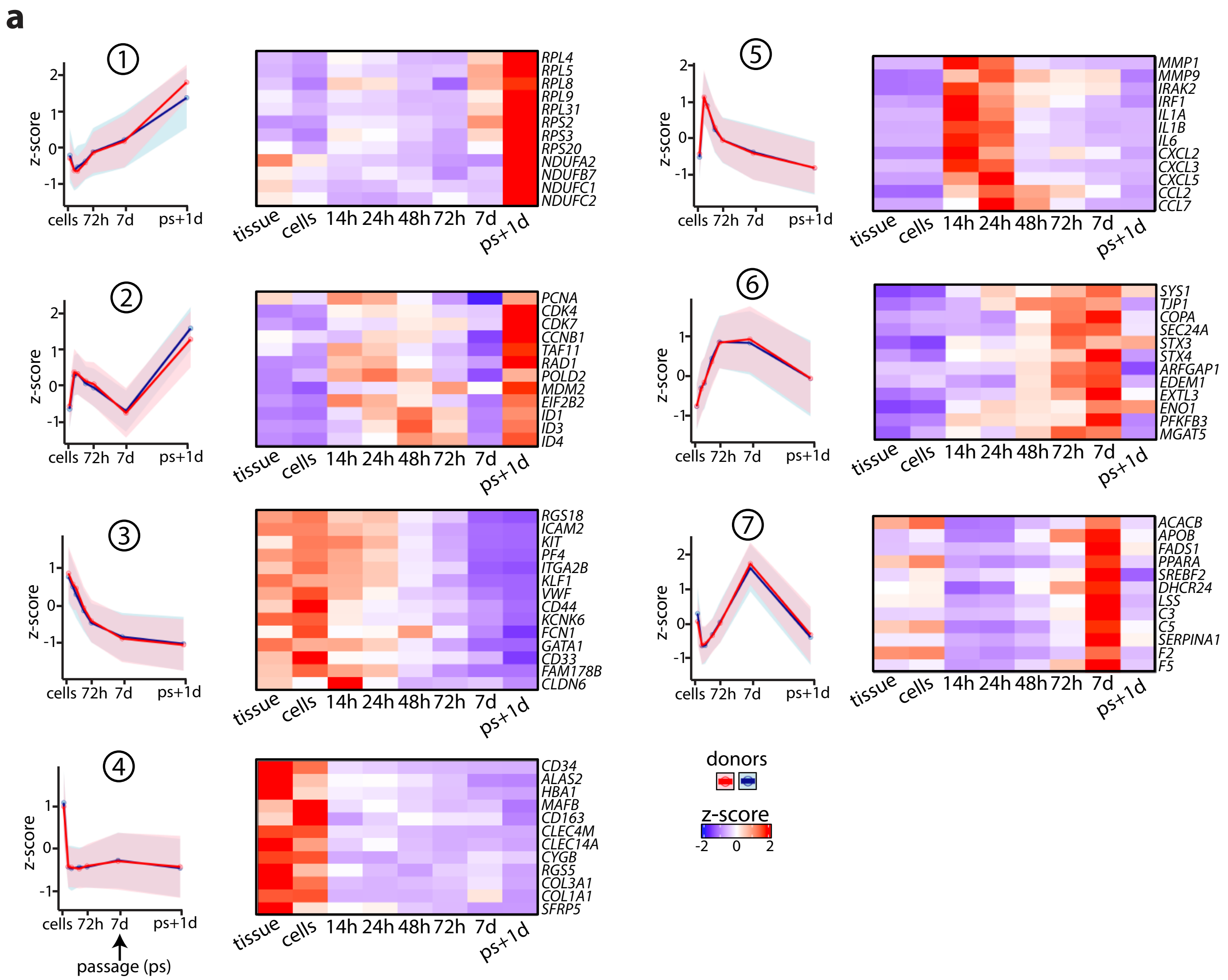

Supplementary Figure 2. Cluster characterization of transcriptomic responses upon human hepatocyte fetal organoid growth from tissue.

a, Heatmaps displaying gene expression patterns of selected genes belonging to the different identified fetal tissue gene clusters. The mean expression patterns of n = 2 donors are visualized as row Z-scores. To the left, the temporal Z-score plots of the different gene clusters presented in **Fig. 1g** are shown for comparative purposes.

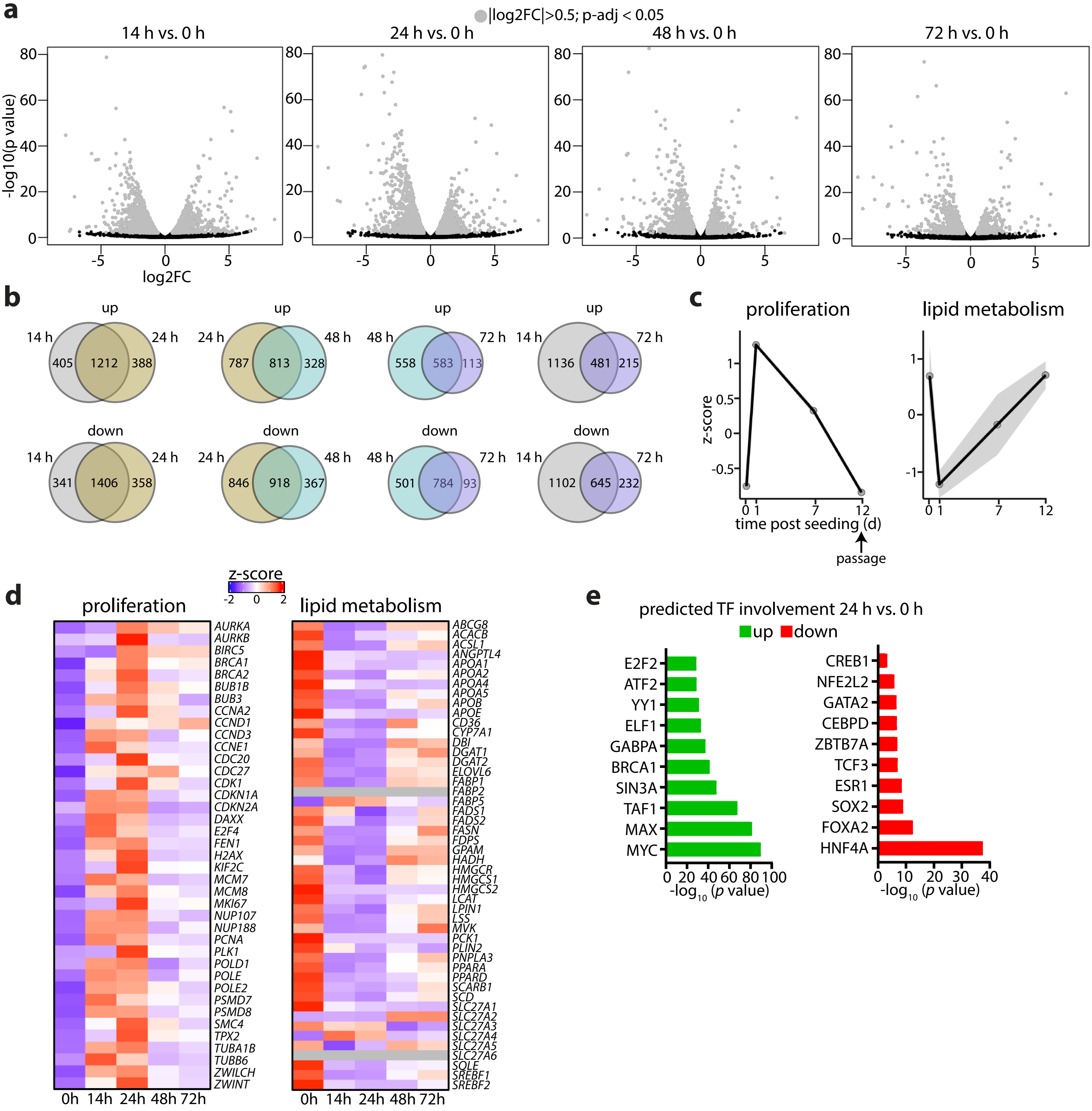

Supplementary Figure 3. Transcriptional responses upon organoid regrowth from single fetal hepatocytes.
a, Volcano plots illustrating differentially expressed genes (|log2FC|>0.5, p-adj < 0.05; grey dots) at the different time points (versus t = 0 h) post seeding of single FHs for organoid formation. Data are derived from n = 2 donors.
b, Venn diagrams illustrating the overlap between the differentially expressed genes at the different timepoints.
c, Temporal Z-score expression of lipid metabolism genes (APOB, MTTP, LSS) and proliferation genes (BUB1B, CCNE1, MCM7) upon organoid growth from single FHs, based on qPCR analysis. At day 12, organoids have fully grown and can be passaged. Mean +- SD expression is plotted, n = 2 organoid cultures.
d, Heatmaps displaying gene expression patterns of proliferation-related genes and lipid metabolism-related genes upon organoid growth from single FHs. The mean expression trends of n = 2 donors are visualized as row Z-scores.
e, GO-term based prediction of involved transcription factors related to the upregulated (green) and downregulated (red) genes at the 24 h timepoint.
Source data are provided as a Source Data file.

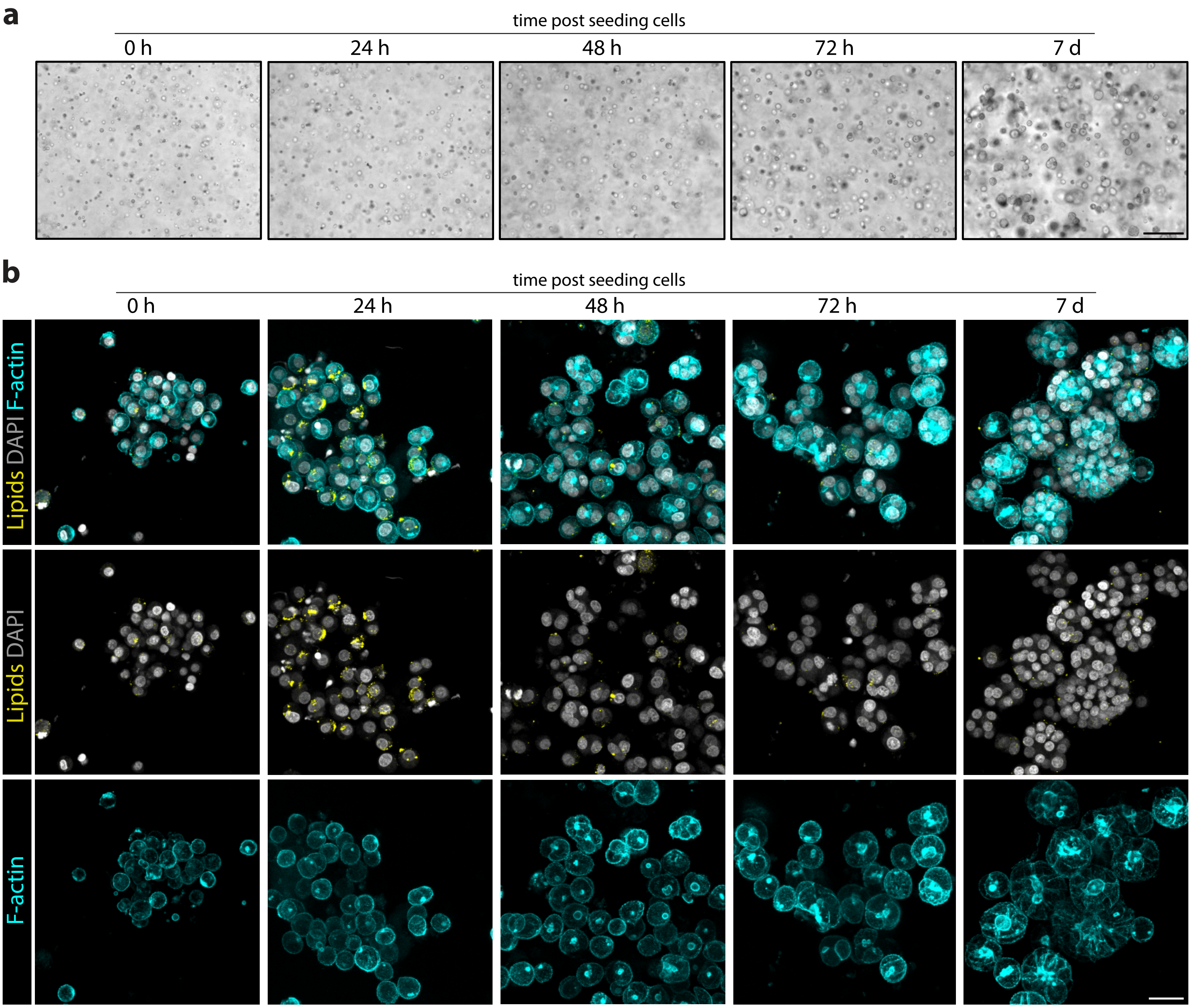

Supplementary Figure 4. Human fetal hepatocytes display transient steatosis during organoid growth.
a-b, Representative low-magnification brightfield images **(a)** and lipid staining (Nile Red) overlaid with phalloidin **(b)** of organoid growth from single FHs over time. Representative of n = 2 outgrowth experiments. Scale bar = 100 μm **(a)** and 50 μm **(b)**.

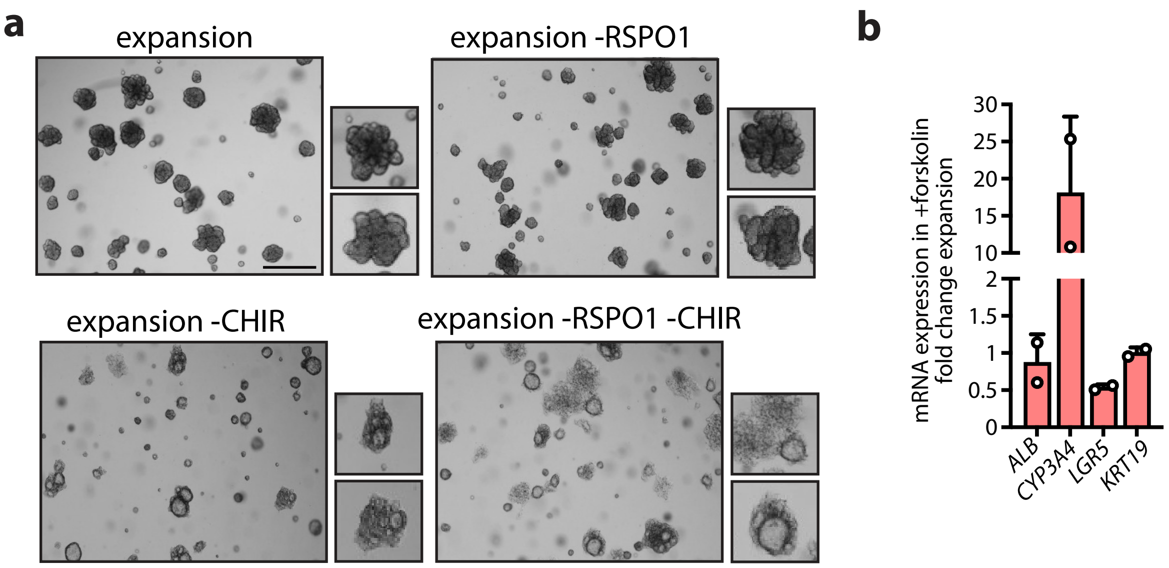

Supplementary Figure 5. Evaluation of removal of Wnt signals from the fetal hepatocyte expansion medium and effect of forskolin exposure.

a, Representative brightfield images of FH organoids in expansion medium or after 14 days of withdrawal of Wnt-related signals. Insets highlight organoid death only occurring upon removal of the GSK3β inhibitor CHIR-99021. Representative of n = 2 medium withdrawal experiments. Scale bar = 400 μm.

b, mRNA expression of ALB, CYP3A4, KRT19, and LGR5 in FH organoids upon addition of the cAMP activator forskolin to the FH expansion medium relative to FH expansion medium without forskolin. Mean +- SD is plotted, n = 2 organoid cultures.

Source data are provided as a Source data file.

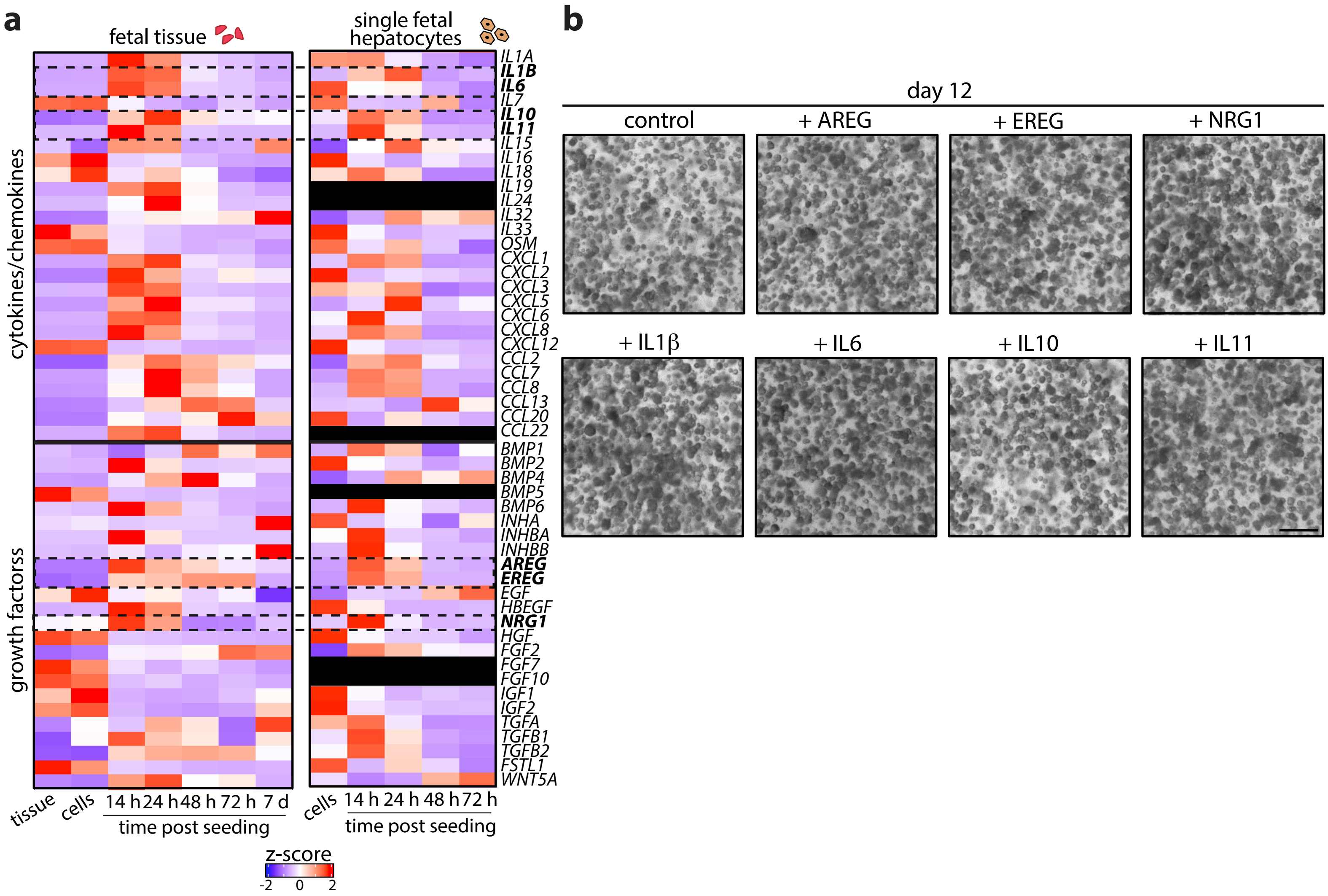

Supplementary Figure 6. Transcriptional dynamics of cytokine and growth factors during human fetal hepatocyte organoid growth and functional validation.

a, Heatmap displaying gene expression patterns of cytokines, chemokines, and growth factors upon organoid growth from single FHs (right) or from tissue (left), the latter as presented in **Fig. 4a**. The mean expression trends of n = 2 donors per condition is visualized as row Z-scores.

b, Representative brightfield images of outgrowing organoids derived from single FHs under the different challenges at day 12. Representative of n = 2 challenge experiments. Scale bar = 100 μm.

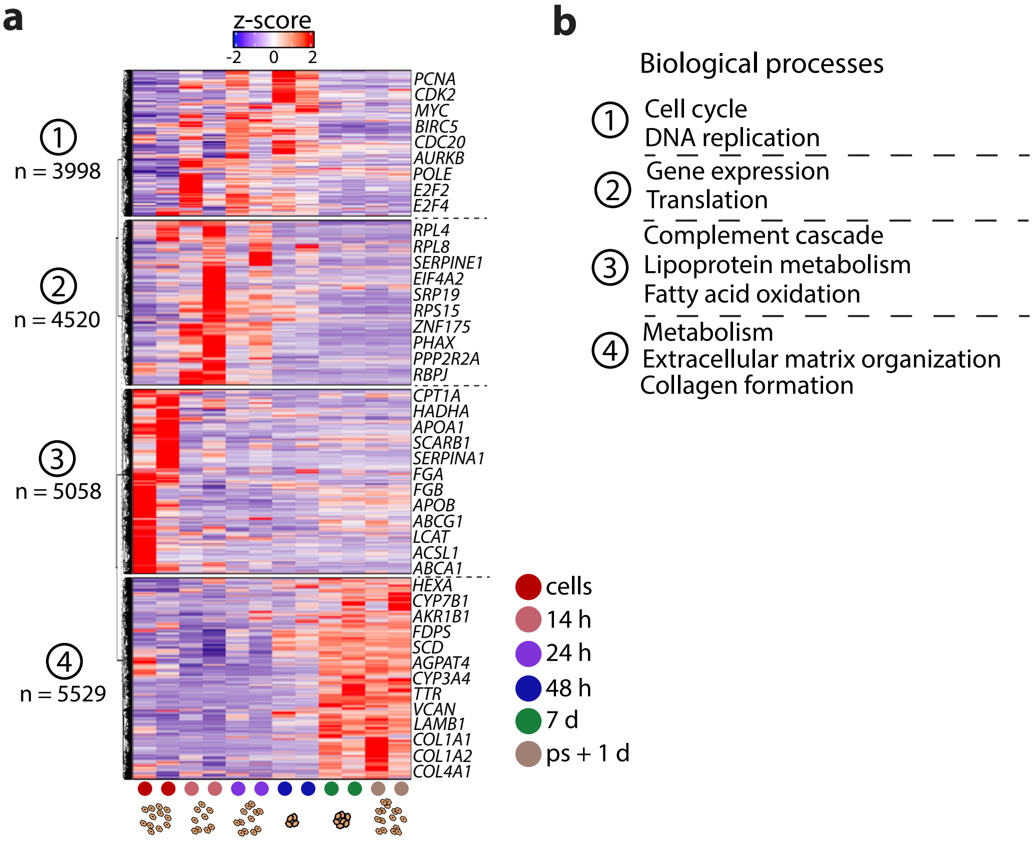

Supplementary Figure 7. Transcriptomic characterization of organoid growth from primary human hepatocytes.

a, Heatmap displaying the temporal expression patterns of genes significantly differently expressed at least at one timepoint versus 0 h ($|\log2FC| > 0.5$, p-adj < 0.05) based on responses of n = 2 donors. The expression patterns are visualized as row Z-scores. The n indicates the number of genes belonging to each cluster.

b, Biological processes associated with the different temporal gene clusters based on GO-term enrichment analysis.

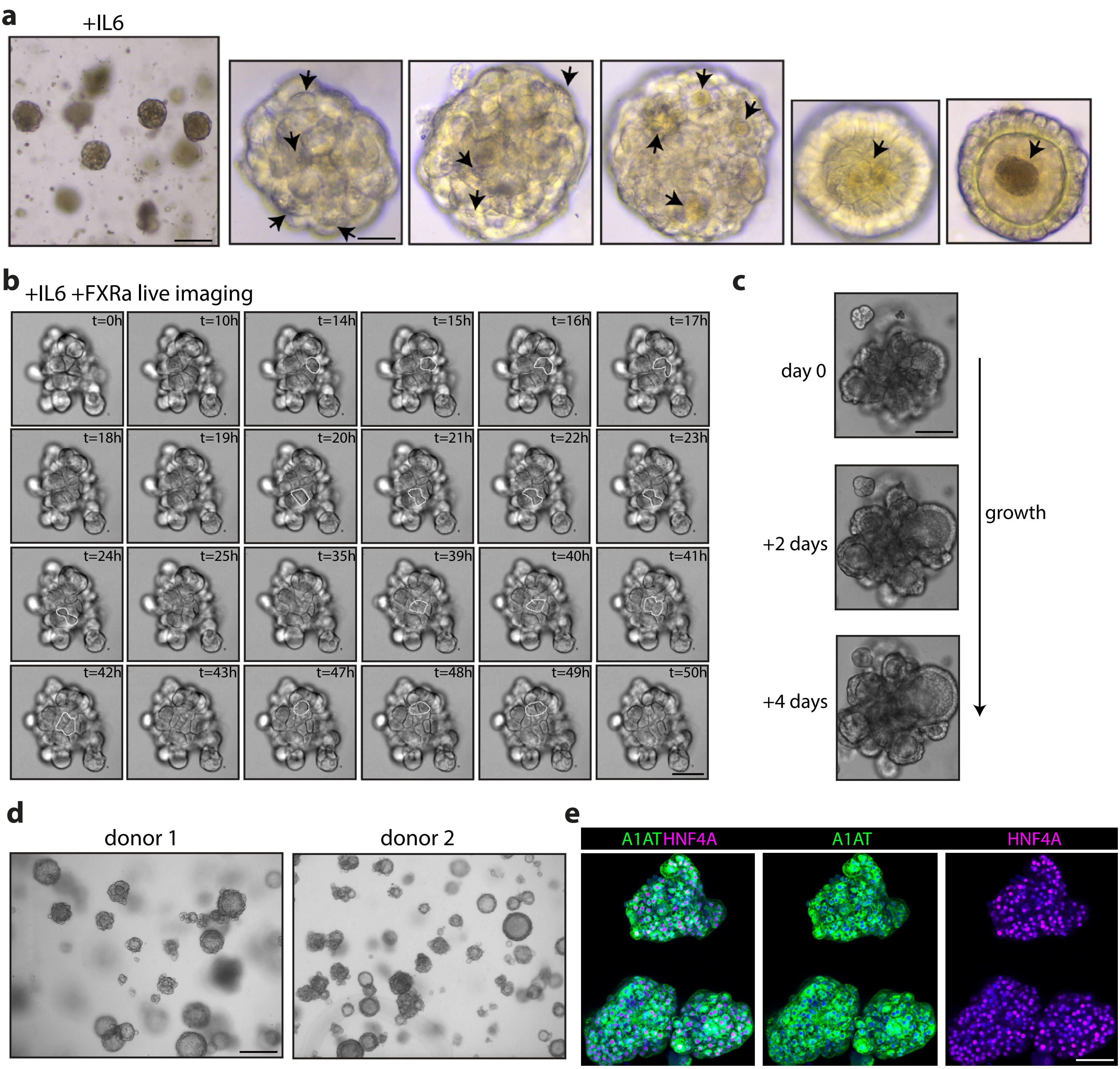

Supplementary Figure 8. IL6+FXRa promotes primary human hepatocyte organoid growth.

a, Representative brightfield images of IL6-cultured PHH organoids, displaying spontaneous lipid accumulation and hints of bile acid accumulation (arrows). Scale bar = 100 μm (low mag) and 20 μm (high mag).

b, Representative time-lapse images visualizing the growth of an IL6+FXRa-cultured PHH organoid (see **Fig. 6f**). White outlines highlight cell divisions. Scale bar = 20 μm.

c, Representative brightfield images of the growth of an IL6+FXRa-cultured PHH organoid over the course of 4 days. Scale bar = 75 μm.

d, Representative brightfield images of PHH organoid cultures from n = 2 donors. Scale bar = 400 μm.

e, Representative low-magnification immunofluorescence staining of PHH organoids for A1AT and HNF4A. Scale bar = 50 μm.

a-e, Representative of characterization of n = 2 expanding PHH organoid cultures.

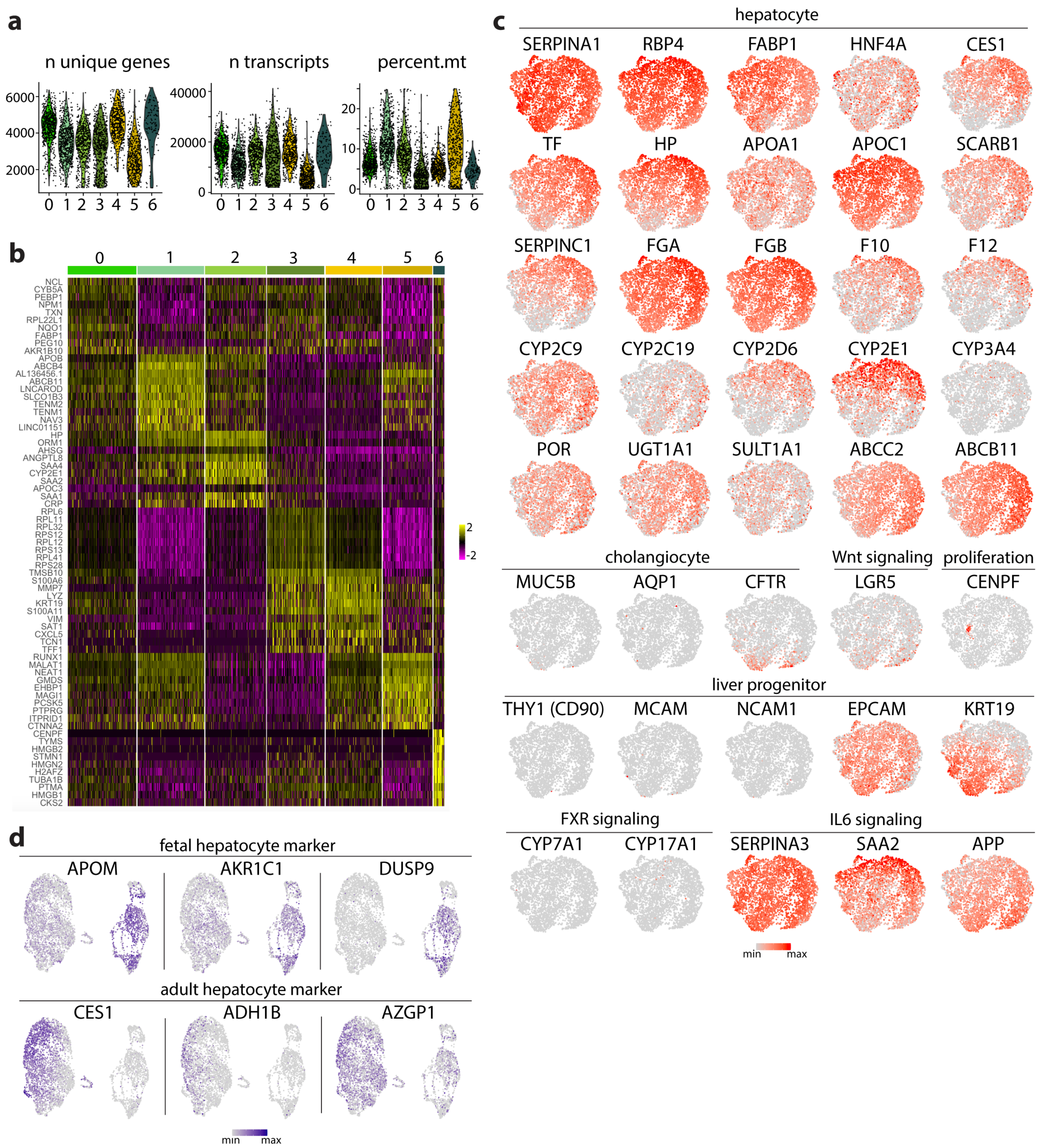

Supplementary Figure 9. Single-cell RNA sequencing analysis of primary human hepatocyte organoids.
a, Data quality measures across the different PHH organoid clusters (see **Fig. 7f**).
b, Heatmap visualizing gene expression trends across clusters of the top 10 genes per cluster.
c, UMAP plots of the indicated markers.
d, UMAP plots of the indicated markers in the combined FH and PHH organoid single-cell RNA sequencing datasets (see **Fig. 7h**).
