# Supplementary material for: Mapping of mitogen and metabolic sensitivity in organoids defines requirements for human hepatocyte growth
Source: Nat Commun. 2024 May 13;15:4034. doi: 10.1038/s41467-024-48550-4 (PMC11091073; doi:10.1038/s41467-024-48550-4)
Supplement: Supplementary file 3 — Description of Additional Supplementary Files [file 41467_2024_48550_MOESM3_ESM.pdf]

File name: Supplementary Data 1
Description: List of qPCR primers used in this study.

File name: Supplementary Movie 1
Description: Live-imaging of IL6- and IL6+FXRa-cultured primary human hepatocyte organoids.

File name: Supplementary Movie 2
Description: Examples of IL6+FXRa-expanding primary human hepatocyte organoid cultures.
