# Supplementary material for: Mapping of mitogen and metabolic sensitivity in organoids defines requirements for human hepatocyte growth
Source: Nat Commun. 2024 May 13;15:4034. doi: 10.1038/s41467-024-48550-4 (PMC11091073; doi:10.1038/s41467-024-48550-4)
Supplement: Supplementary file 7 — Reporting Summary [file 41467_2024_48550_MOESM7_ESM.pdf]

nature portfolio

Corresponding author(s): Delilah Hendriks, Benedetta Artegiani, Hans Clevers

Last updated by author(s): Apr 19, 2024

Reporting Summary

Nature Portfolio wishes to improve the reproducibility of the work that we publish. This form provides structure for consistency and transparency in reporting. For further information on Nature Portfolio policies, see our Editorial Policies and the Editorial Policy Checklist.

Statistics

For all statistical analyses, confirm that the following items are present in the figure legend, table legend, main text, or Methods section.

n/a	Confirmed	
☐	☒	The exact sample size (*n*) for each experimental group/condition, given as a discrete number and unit of measurement
☐	☒	A statement on whether measurements were taken from distinct samples or whether the same sample was measured repeatedly
☐	☒	The statistical test(s) used AND whether they are one- or two-sided<br>*Only common tests should be described solely by name; describe more complex techniques in the Methods section.*
☒	☐	A description of all covariates tested
☐	☒	A description of any assumptions or corrections, such as tests of normality and adjustment for multiple comparisons
☐	☒	A full description of the statistical parameters including central tendency (e.g. means) or other basic estimates (e.g. regression coefficient) AND variation (e.g. standard deviation) or associated estimates of uncertainty (e.g. confidence intervals)
☐	☒	For null hypothesis testing, the test statistic (e.g. *F*, *t*, *r*) with confidence intervals, effect sizes, degrees of freedom and *P* value noted<br>*Give P values as exact values whenever suitable.*
☒	☐	For Bayesian analysis, information on the choice of priors and Markov chain Monte Carlo settings
☒	☐	For hierarchical and complex designs, identification of the appropriate level for tests and full reporting of outcomes
☒	☐	Estimates of effect sizes (e.g. Cohen's *d*, Pearson's *r*), indicating how they were calculated

Our web collection on statistics for biologists contains articles on many of the points above.

Software and code

Policy information about availability of computer code

Data collection	Leica LAS X (v3.5.7)
Data analysis	ImageJ (Fiji) (v2.14.0), GraphPad Prism (v9.4.1), DESeq2 (v1.36.0), Seurat (v4.3.0), souporcell (v2.0), Enrichr

For manuscripts utilizing custom algorithms or software that are central to the research but not yet described in published literature, software must be made available to editors and reviewers. We strongly encourage code deposition in a community repository (e.g. GitHub). See the Nature Portfolio guidelines for submitting code & software for further information.

Data

Policy information about availability of data

All manuscripts must include a data availability statement. This statement should provide the following information, where applicable:

- Accession codes, unique identifiers, or web links for publicly available datasets
- A description of any restrictions on data availability
- For clinical datasets or third party data, please ensure that the statement adheres to our policy

Read-level bulk and single-cell RNA sequencing data generated in this study have been deposited in the GEO database under accession code “GSE264262 https://www.ncbi.nlm.nih.gov/geo/query/acc.cgi?acc=GSE264262”. The associated raw data are protected and are not available due to data privacy laws. Source data are provided with this paper.

Research involving human participants, their data, or biological material

Policy information about studies with human participants or human data. See also policy information about sex, gender (identity/presentation), and sexual orientation and race, ethnicity and racism.

Reporting on sex and gender	Findings in this study were performed using human fetal liver tissue from both sexes, i.e. males (XX) and females (XY), determined by karyotyping. Sex and gender were not considered in this study design.
Reporting on race, ethnicity, or other socially relevant groupings	Race, ethnicity, and other socially relevant groupings were not considered and are not reported on in this study design.
Population characteristics	Anonymized healthy human fetal livers from both males and females were included.
Recruitment	We included in the study any available anonymously donated healthy human fetal liver tissues upon informed consent. No pre-selection was performed, to avoid any bias.
Ethics oversight	Anonymized human fetal livers became available after pregnancy terminations and upon informed consent and under ethical permission from the Leiden University Medical Center.

Note that full information on the approval of the study protocol must also be provided in the manuscript.

Field-specific reporting

Please select the one below that is the best fit for your research. If you are not sure, read the appropriate sections before making your selection.

[x] Life sciences   [ ] Behavioural & social sciences   [ ] Ecological, evolutionary & environmental sciences

For a reference copy of the document with all sections, see nature.com/documents/nr-reporting-summary-flat.pdf

Life sciences study design

All studies must disclose on these points even when the disclosure is negative.

Sample size	No statistical method was used to predetermine sample size. Transcriptomic profiling was performed using n = 2 donors, which is a standard sample size in the field to describe transcriptomic trends. The other results were validated across multiple donors and using multiple organoids and experiments. Exact details on the n are indicated in the figure legends.
Data exclusions	No data were excluded from the analyses.
Replication	Experiments were replicated at least 2 times. Experiments were confirmed using organoid cultures from multiple (at least 2) donors. All attempts at replication were successful.
Randomization	The experiments were not randomized, since no experiments required randomization for analysis in the experimental set-up of the study.
Blinding	The investigators were not blinded to allocation during experiments and outcome assessment, since no experiments required blinding for analysis in the experimental set-up of the study.

Reporting for specific materials, systems and methods

We require information from authors about some types of materials, experimental systems and methods used in many studies. Here, indicate whether each material, system or method listed is relevant to your study. If you are not sure if a list item applies to your research, read the appropriate section before selecting a response.

Materials & experimental systems

n/a	Involved in the study	
[ ]	[x]	Antibodies
[ ]	[x]	Eukaryotic cell lines
[x]	[ ]	Palaeontology and archaeology
[x]	[ ]	Animals and other organisms
[x]	[ ]	Clinical data
[x]	[ ]	Dual use research of concern
[x]	[ ]	Plants

Methods

n/a	Involved in the study	
[x]	[ ]	ChIP-seq
[x]	[ ]	Flow cytometry
[x]	[ ]	MRI-based neuroimaging

Antibodies

Antibodies used	Anti-Ki67 #14-5698-82 (SolA15), Thermo Fisher dilution 1 t,o 1000; Phalloidin-Atto 647N Sigma-Aldrich #65906 dilution 1 to 1000; Anti-beta catenin (H-102) #sc-7199 Santa Cruz dilution 1 to 1000; Anti-ZO1 #PA5-19090 Thermo Fisher, dilution 1 to 500; Anti-ALB #A80-229A Thermo-Fisher dilution 1 to 300; Anti-KRT7 Thermo Fisher MA5-11986 clone OV-TL 12/30 dilution 1 to 400, Anti-KRT19 13092S (D7F7W) Cell Signalling Technology dilution 1 to 500, anti-A1AT rabbit polyclonal 1:500 (Abcam, #ab9373), anti-AFP rabbit polyclonal 1:250 (Thermo Fisher Scientific, #PAS-16658), Alexa- Fluor 488 anti-rabbit #A21206, Alexa-Fluor 647 anti-rat #A21247, Alexa-Fluor 488 anti-rabbit #A21206, Alexa-Fluor 568 anti-mouse #A10037, Alexa-Fluor 488 anti-goat #A11055.
Validation	Anti-Ki-67 purified (SolA15) #14-5698-82 (https://www.thermofisher.com/antibody/product/Ki-67-Antibody-clone-SolA15-Monoclonal/14-5698-82). This Antibody was verified by Cell treatment to ensure that the antibody binds to the antigen stated. Citations: e.g. PMID: 34525348, PMID: 34100459.<br><br>Phalloidin-Atto 647 #65906 (https://www.sigmaaldrich.com/NL/en/product/sigma/65906).Citations; e.g. PMID32123335.<br><br>Anti-beta catenin #sc-7199 (https://www.scbt.com/p/beta-catenin-antibody-h-102) The use of this antibody has been validated in several publications e.g. PMID: 28793266, PMID: 25645929<br><br>Anti-ALB #A80-229A (https://www.thermofisher.com/antibody/product/Human-Albumin-Antibody-Polyclonal/A80-229A). By immunoelectrophoresis and ELISA this antibody reacts specifically with human albumin. Less than 0.1% cross reactivity to bovine, mouse and pig albumin was detected. Citations: e.g. PMID: 30500538.<br><br>Anti-AFP #PA5-16658 (https://www.fishersci.com/shop/products/afp-polyclonal-antibody-invitrogen-2/PIPA516658). TAFP Polyclonal antibody specifically detects AFP in Human, Ovine, Porcine samples. It is validated for Immunocytochemistry, Immunohistochemistry (Paraffin), Western Blot. Citations: e.g. PMID: 32123335.<br><br>Anti-MRP2 #ab3343 (M2 III-6) (https://www.abcam.com/en-nl/products/primary-antibodies/mrp2-antibody-m2-iii-6-ab3373). Validated in WB and tested in Human samples. Cited in 72 publications e.g. PMID: 11016657.<br><br>Anti-ZO1 #PA5-19090 (https://www.thermofisher.com/antibody/product/PA5-19090.html?ef_id=Cj0KCQjw2cWgBhDYARIsALggUhqMYTVZX8hpXnjKbUTy6Ao3d8M31mNUy7Wz7lcVpQA-ymyyw60wHN4aAkZZEALw_wcB:G:s&s_kwcid=AL!3652!3!459737518508!!!g!!!10950825775!106531320406&cid=bid_pca_aup_r01_co_cp1359_pjt0000_bid00000_0se_gaw_dy_pur_con&gclid=Cj0KCQjw2cWgBhDYARIsALggUhqMYTVZX8hpXnjKbUTy6Ao3d8M31mNUy7Wz7lcVpQA-ymyyw60wHN4aAkZZEALw_wcB), This Antibody was verified by Independent antibody to ensure that the antibody binds to the antigen stated and used in several publications e.g. PMID: 32403233.<br><br>Anti-KRT7 #MA5-11986 (https://www.thermofisher.com/antibody/product/Cytokeratin-7-Antibody-clone-OV-TL-12-30-Monoclonal/MA5-11986MA5-11986 clone OV-TL 12/30). This Antibody was verified by Relative expression to ensure that the antibody binds to the antigen stated.<br><br>Anti-KRT19 #13092S (D7F7W) Cell Signalling Technology (https://www.cellsignal.com/products/primary-antibodies/keratin-19-antibody/3479). The use of this antibody has been validated in several previous publications, e.g. PMID: 33247284, PMID: 32123335

Eukaryotic cell lines

Policy information about cell lines and Sex and Gender in Research

Cell line source(s)	Hepatocyte organoid lines were generated from human fetal liver tissues from termination material from donors with informed consent under ethical permission (Leiden University Medical Center) and from primary human hepatocytes obtained from commercial sources.
Authentication	The organoid lines were not authenticated.
Mycoplasma contamination	Organoid lines were monthly tested for mycoplasma contamination and tested negative without exception.
Commonly misidentified lines<br>(See ICLAC register)	No commonly misidentified cell lines were used.
